# Supplementary material for: RNF208, an estrogen-inducible E3 ligase, targets soluble Vimentin to suppress metastasis in triple-negative breast cancers
Source: Nat Commun. 2019 Dec 20;10:5805. doi: 10.1038/s41467-019-13852-5 (PMC6925134; doi:10.1038/s41467-019-13852-5)
Supplement: Supplementary file 1 — Supplementary Information [file 41467_2019_13852_MOESM1_ESM.pdf]

Supplementary Information

RNF208, an estrogen-inducible E3 ligase, targets soluble Vimentin to suppress metastasis in triple-negative breast cancers

Kyoungwha Pang, Jinah Park, Sung Gwe Ahn, Jihee Lee, Yuna Park, Akira Ooshima, Seiya Mizuno, Satoshi Yamashita, Kyung-Soon Park, So-Young Lee, Joon Jeong, Toshikazu Ushijima, Kyung-Min Yang, and Seong-Jin Kim

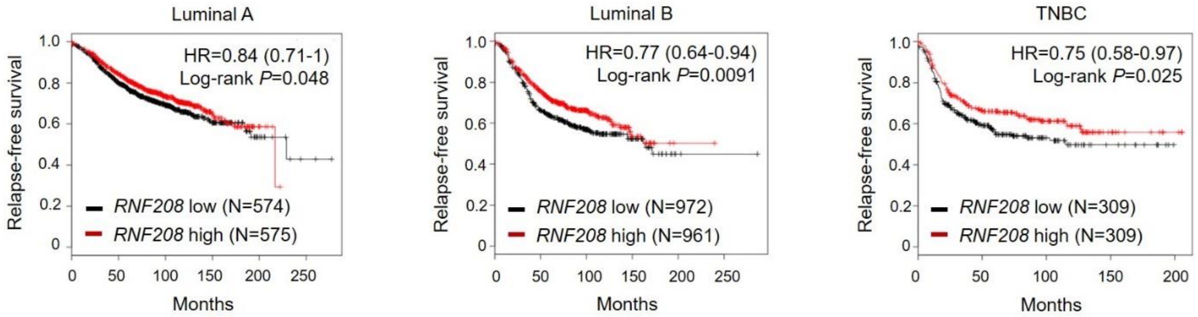

Supplementary Figure 1 Underexpression of RNF208 is correlated with poor clinical outcome. Kaplan-Meier analysis showing the association between RNF208 expression and relapse-free survival according to breast cancer subtypes using public meta-analyses (Luminal A N=1,149, Luminal B N=1,933, TNBC N=618 patients). *P* values were calculated using a log-rank test.

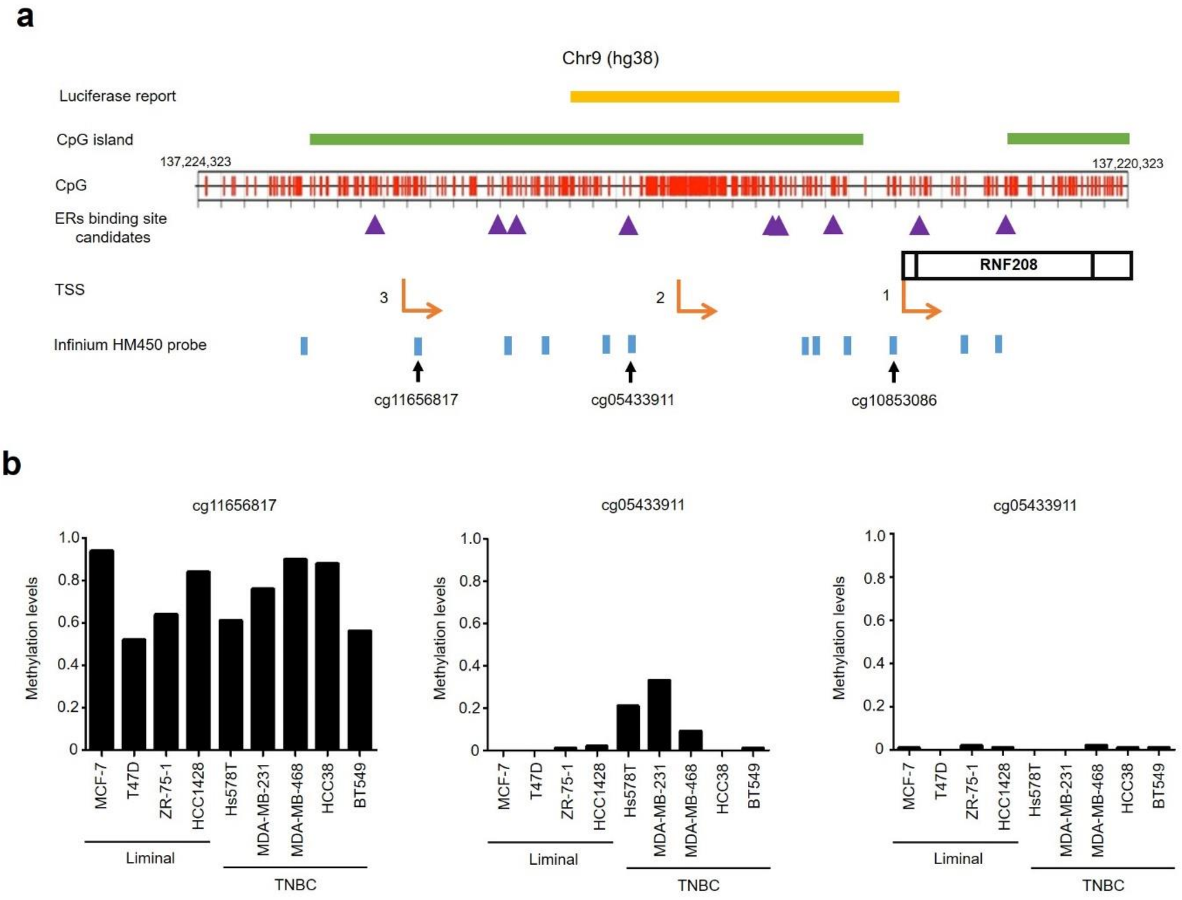

Supplementary Figure 2 Underexpression of *RNF208* is not associated with DNA methylation in breast cancer cells. (**a**) Genomic structure of *RNF208*. Transcriptional start sites (TSSs) of *RNF208* were identified using the mRNA sequences in the GenBank and a TSS database (DBTSS, https://dbtss.hgc.jp/). The fragment used for the luciferase assay is shown by the orange box. (**b**) Methylation levels of the three TSSs of *RNF208*. Beta values of CpGs close to the TSSs were obtained from a database (GEO, https://www.ncbi.nlm.nih.gov/geo/, GSE68379). The *RNF208* expression levels in nine breast cancer cell lines were not associated with methylation levels of any of the three TSSs.

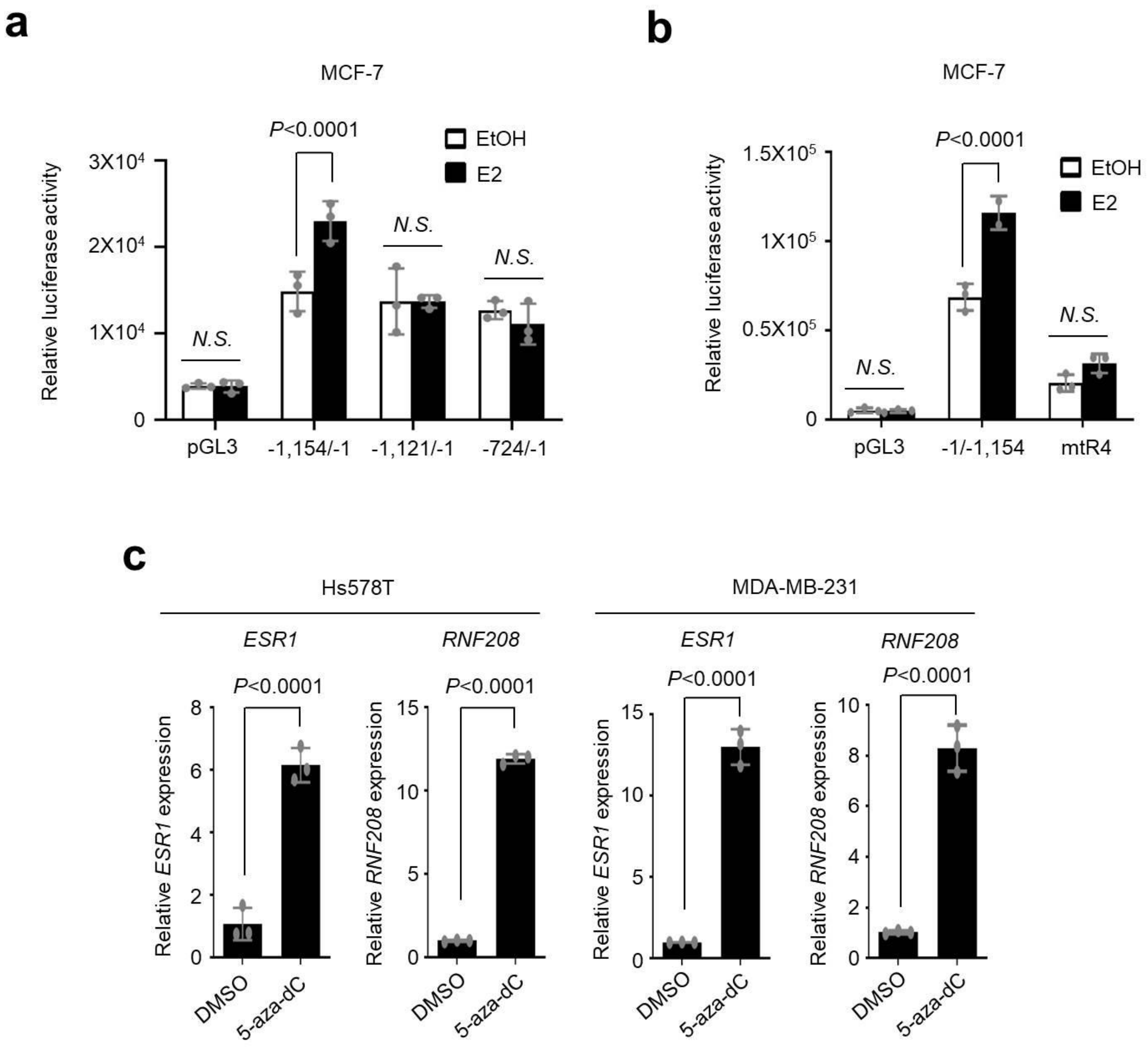

Supplementary Figure 3 ERα expression is required for the induction of RNF208 expression. (**a**) MCF-7 cells were transfected with various deletion constructs of the RNF208 promoter and then treated with or without 10 nM of E2 for 24 h. After E2 treatment, cells were assayed for luciferase activity. Error bars indicate ±S.D.M. of three independent experiments. (**b**) MCF-7 cells were transfected with pGL3 control, RNF208 promoter, or its mutant (mtR4) plasmids containing mutated estrogen responsive element site (CACC sequence replaced by GAAA) and then subjected to luciferase assays. (**c**) qRT-PCR of *ESR1* and *RNF208* expression in 5-aza-dC-treated TNBC cells. Hs578T and MDA-MB-231 cells were treated with 10 μM 5-aza-dC for 24 h. *18s* was used as internal control. All *P* values were calculated by unpaired two-tailed Student's *t*-tests. All data represent means ±S.D. of three independent experiments. Source data for **a-c** are available in Supplementary Table 4.

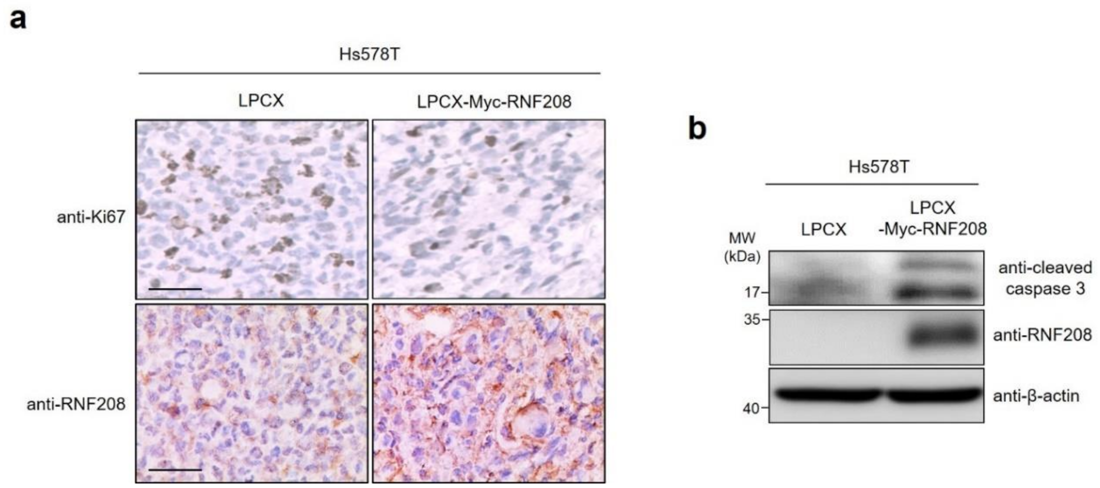

Supplementary Figure 4 Overexpression of RNF208 reduces tumorigenesis in TNBC cells. (**a**) Representative IHC image showing Ki-67 and RNF208 expression in primary tumor tissues from **main Fig. 3b**. Original magnification 100×. Scale bar, 100 μm. (**b**) Immunoblot analysis of active caspase-3 expression using lysates of RNF208-overexpressing Hs578T cells. β-actin was used as an internal control. Unprocessed original scans of blots in **b** are shown in Supplementary Fig. 13.

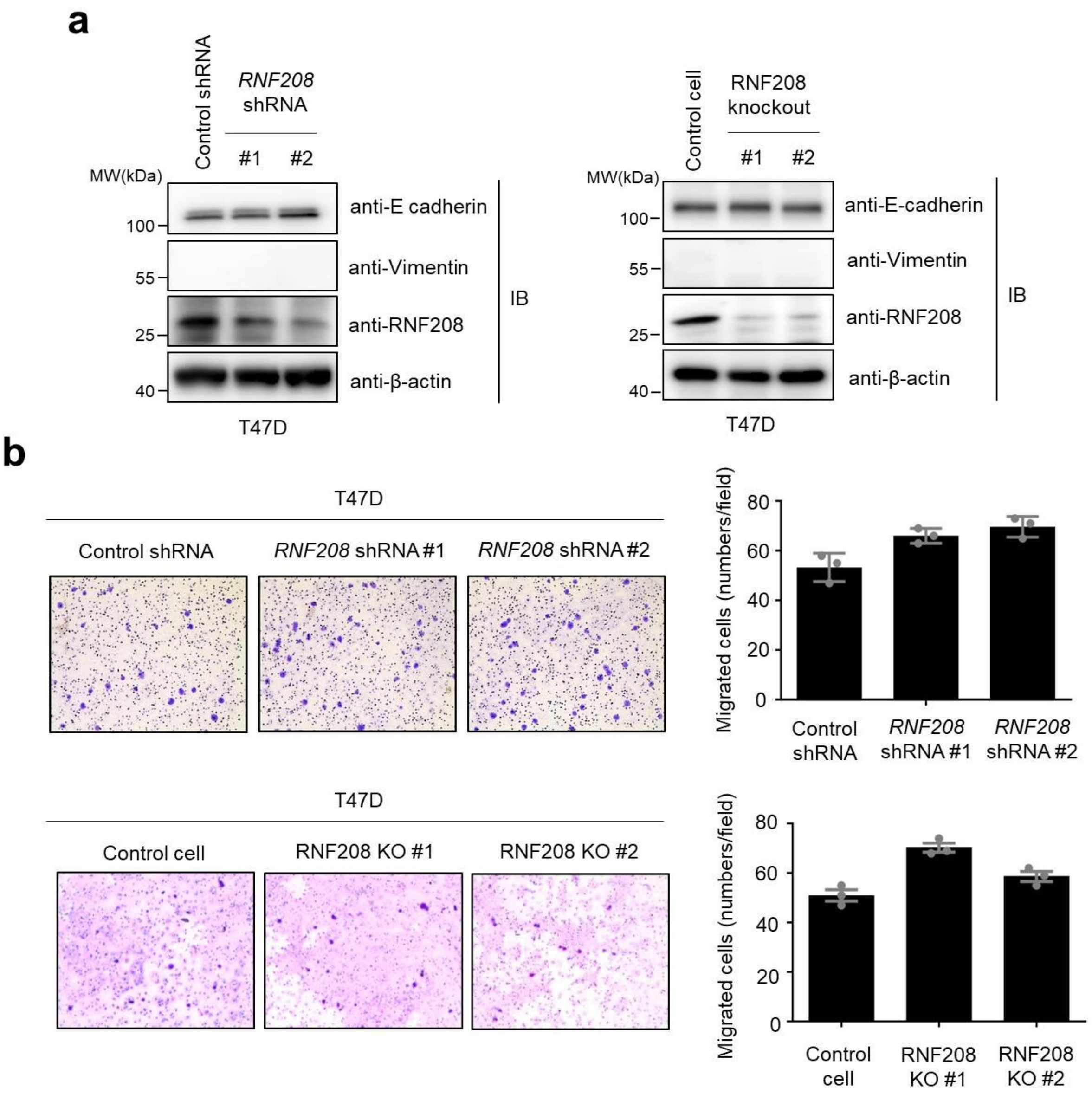

Supplementary Figure 5 Loss of RNF208 does not influence aggressive phenotype-induced cancer progression in luminal breast cancer subtypes. (**a**) Immunoblot analysis showing E-cadherin and Vimentin expression in RNF208 knockdown and knockout T47D cells. (**b**) Transwell migration assays of T47D cells stably expressing *RNF208* shRNAs or RNF208 knockout T47D cells. Migrated cells were counted following staining with crystal violet. The data represent means ±S.D. of three independent experiments. Source data for **b** is available in Supplementary Table 4. Unprocessed original scans of blots in **a** are shown in Supplementary Fig. 13.

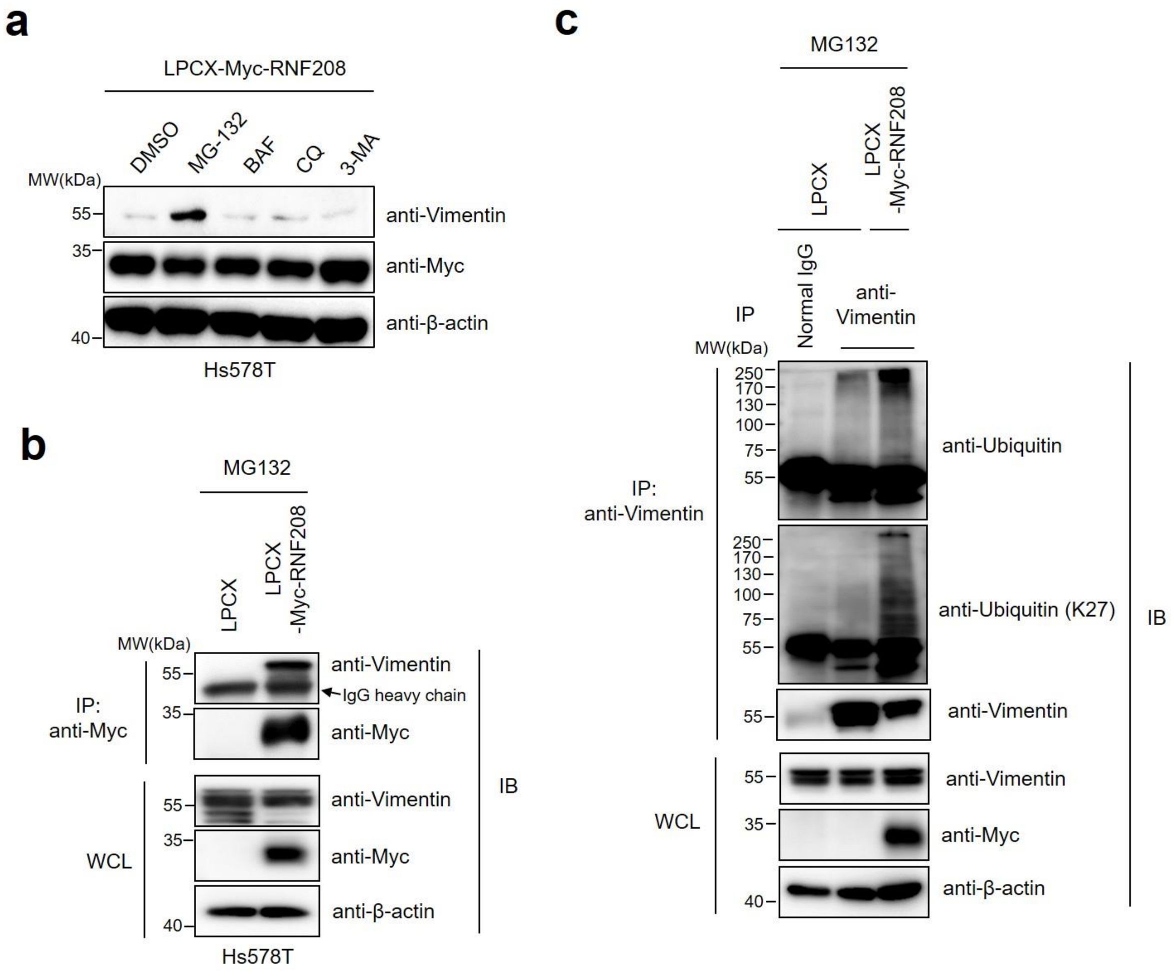

Supplementary Figure 6 RNF208 induces the proteasomal degradation of vimentin through interaction between RNF208 and Vimentin. (**a**) RNF208-overexpressing Hs578T cells were treated with 10 μM MG132, 100 nM bafilomycin A1 (BAF), 20 μM chloroquine (CQ), and 10 mM 3-methyladenine (3-MA) for 6 h. Cell lysates were immunoblotted with the indicated antibodies. (**b**) Immunoprecipitation assay showing endogenous interaction between RNF208 and Vimentin in RNF208-overexpressing Hs578T cells upon MG132 treatment. Cell lysates were immunoprecipitated with anti-Myc antibody and immunoblotted with the indicated antibodies. (**c**) RNF208 overexpression induces the endogenous K27-linked ubiquitination of Vimentin. Lysates of control or RNF208-overexpressing MDA-MB-231 cells were immunoprecipitated with anti-Vimentin antibody and then immunoblotted with the indicated antibodies. Unprocessed original scans of blots in **a-c** are shown in Supplementary Fig. 13.

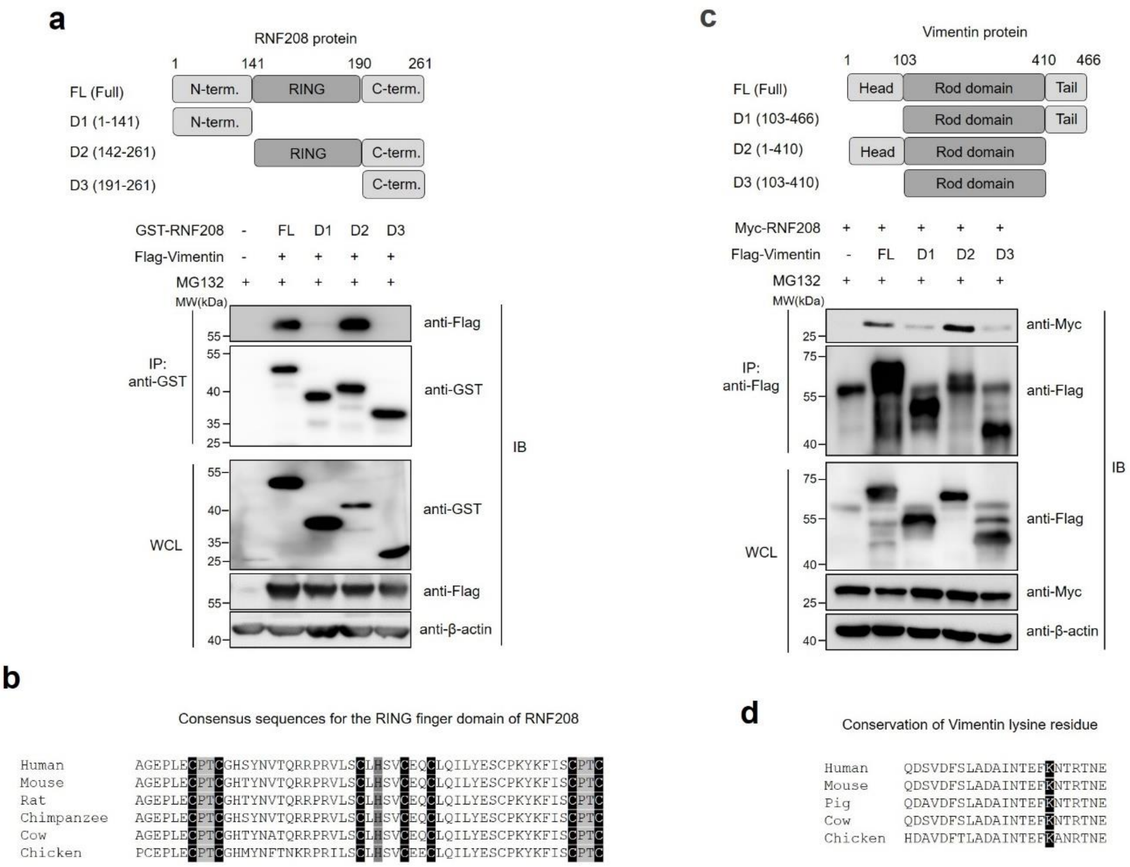

Supplementary Figure 7 RING domain of RNF208 specifically interacts with head domain of Vimentin. (**a**) Immunoprecipitation assay in 293T cells co-transfected with Flag-Vimentin plasmids with GST-wild-type RNF208 (FL) or GST-RNF208 deletion mutants (1-141 a.a., D1; 142-261 a.a., D2; 191-261 a.a., D3) upon MG132 treatment. Cell lysates were immunoprecipitated with Glutathione Sepharose beads and immunoblotted with the indicated antibodies. (**b**) Consensus sequence for the RING finger domain of RNF208 in different species. (**c**) Immunoprecipitation assay in 293T cells co-transfected with Myc-RNF208 plasmids with Flag-wild-type Vimentin or Flag-Vimentin deletion mutants (103-466 a.a., D1; 1-410 a.a., D2; 103-410 a.a., D3) upon MG132 treatment. Cell lysates were immunoprecipitated with anti-Flag antibody and immunoblotted with the indicated antibodies. (**d**) Conservation of Vimentin at Lys97 residue in diverse species. Unprocessed original scans of blots in **a**,**c** are shown in Supplementary Fig. 13.

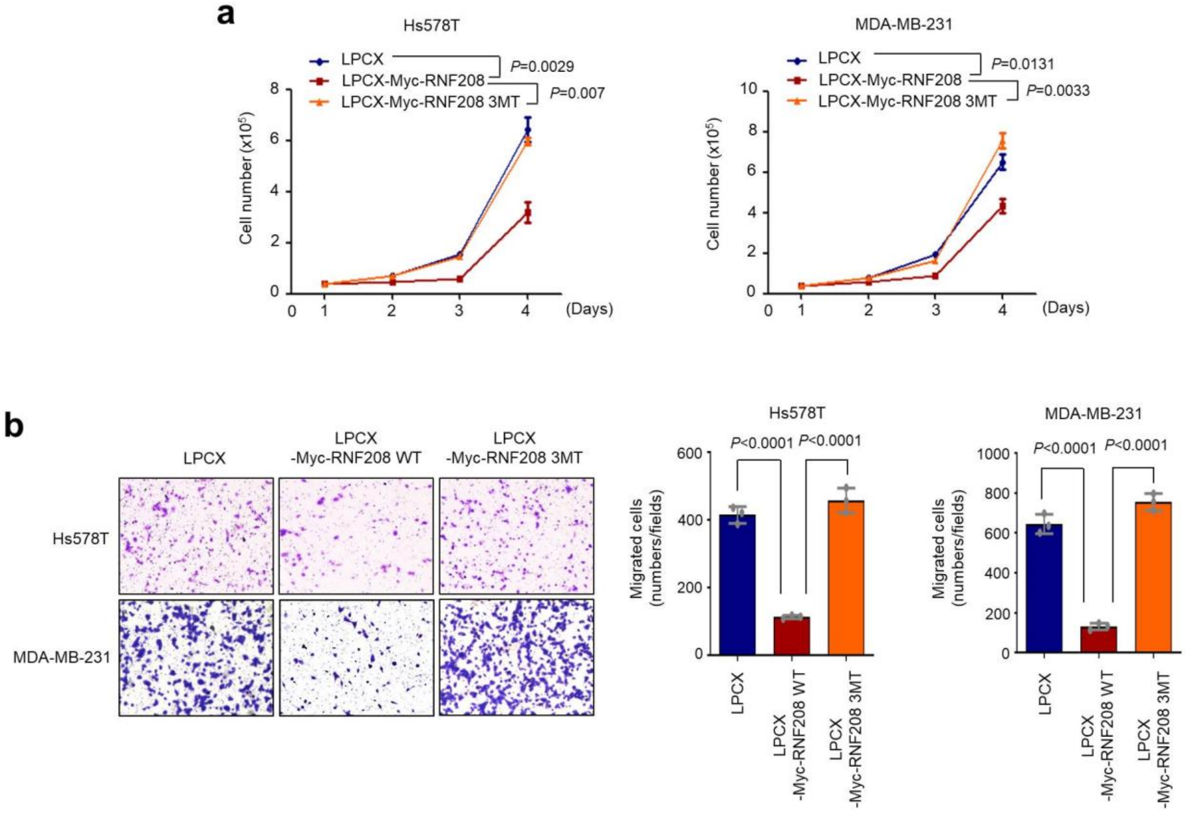

Supplementary Figure 8 Activity of RNF208 E3 ligase regulates the cell proliferation and migration of TNBC cells. (**a**) Cell doublings of TNBC cells stably expressing wild-type RNF208 or RNF208 (3MT) mutant proteins. Each point represents the mean of cell numbers counted in triplicate dishes. (**b**) Transwell migration assays of wild-type RNF208 or RNF208 (3MT) mutant-overexpressing TNBC cells. Migrated cells were counted following staining with crystal violet (upper) or toluidine blue (bottom). All *P* values were calculated by unpaired two-tailed Student's *t*-tests. All data represent means ± S.D. of three independent experiments. Source data for **a**,**b** are available in Supplementary Table 4.

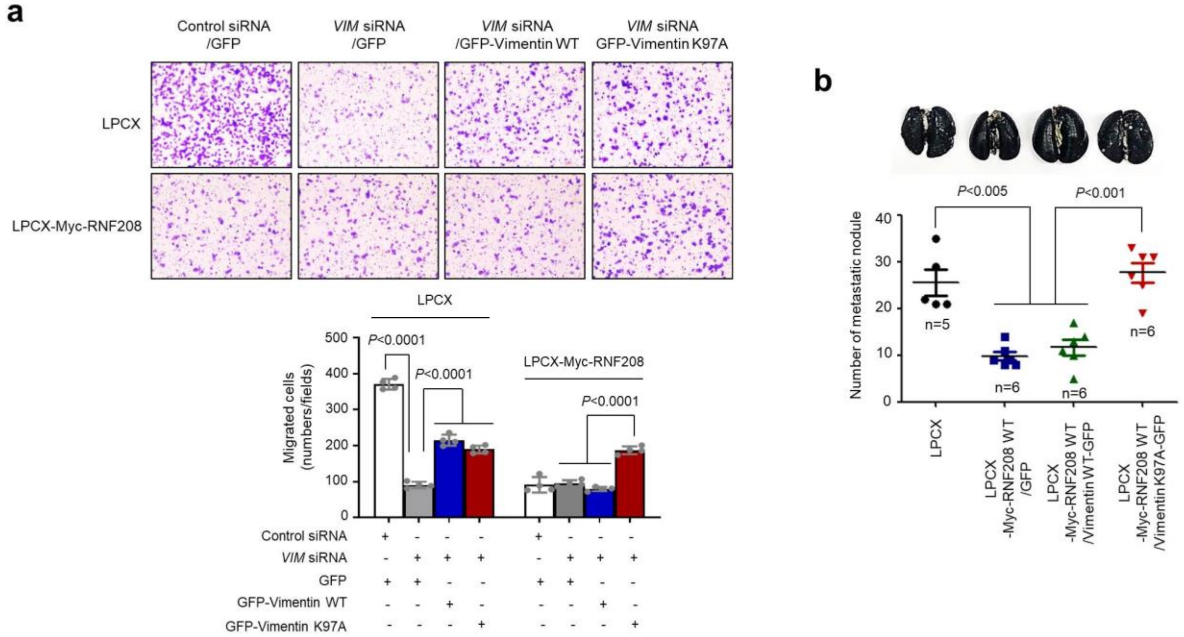

Supplementary Figure 9 RNF208 suppresses metastasis by targeting the Lys97 residue of Vimentin. (**a**) Transwell migration assays by RNF208 overexpression in Vimentin-rescued MDA-MB-231 cells. Cells were transfected with specific *VIM* siRNA, followed by transfection with GFP-Vimentin WT or K97A mutant plasmids, respectively. Migrated cells were counted following staining with crystal violet. (**b**) Representative whole lung image stained with India ink showing metastatic nodules from 8 weeks, derived from lateral tail-vein injection of control, RNF208 WT, RNF208 WT/Vimentin WT or RNF208 WT/Vimentin (K97A) mutant-overexpressing MDA-MB-231cells (upper). Scatter plot showing the number of lung metastatic nodules (bottom) (n=6 per group). All *P* value was calculated by unpaired two-tailed Student's *t*-tests. All data represent the mean ±S.D. Source data for **a**,**b** are available in Supplementary Table 4.

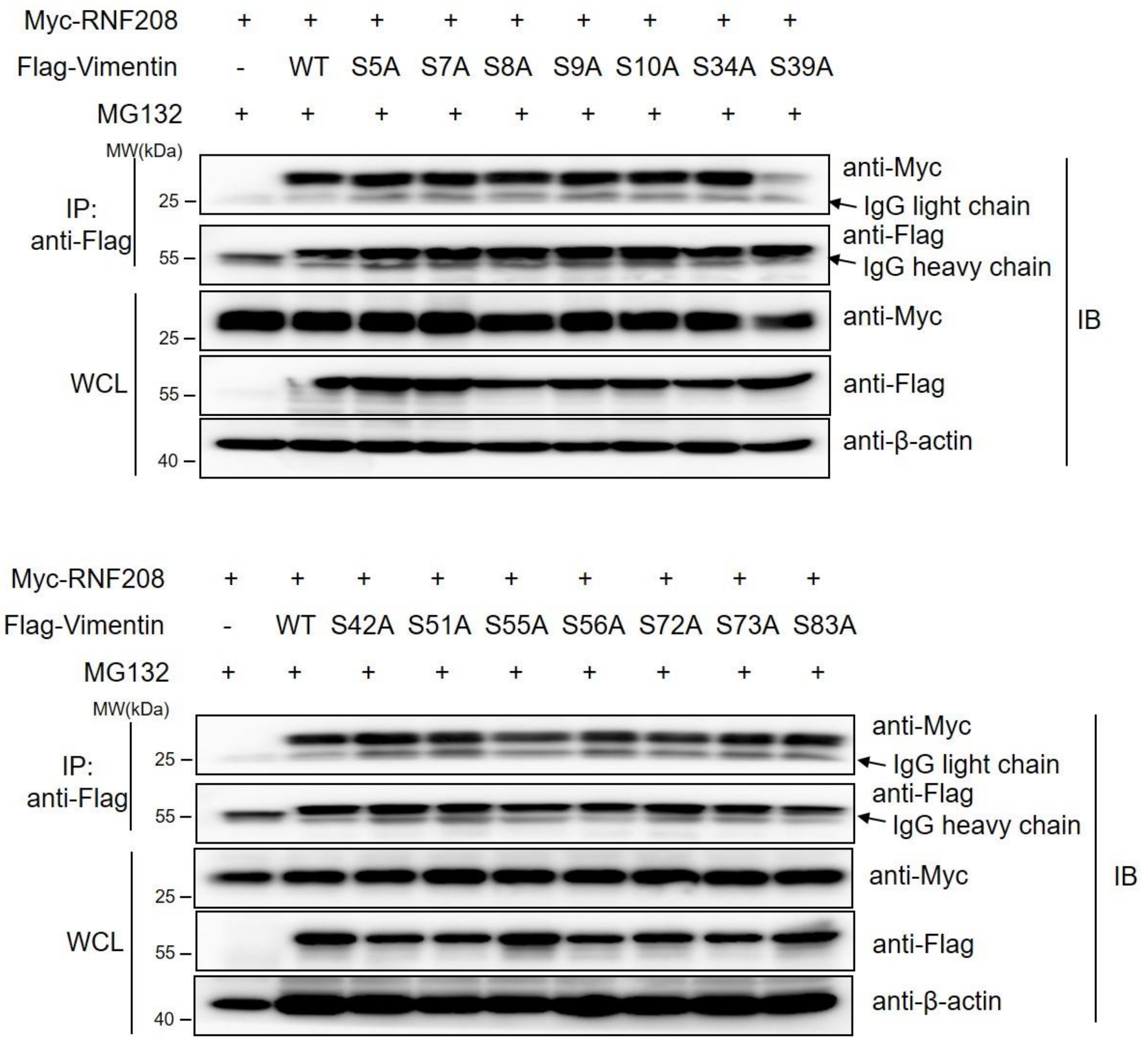

Supplementary Figure 10 RNF208 specifically interacted with Vimentin at the Ser39 residue. The 293T cells were co-transfected with Myc-RNF208 and various Flag-Vimentin mutant plasmids upon MG132 treatment. Cell lysates were immunoprecipitated with anti-Flag antibody and then immunoblotted with the indicated antibodies. Unprocessed original scans of blots are shown in Supplementary Fig. 13.

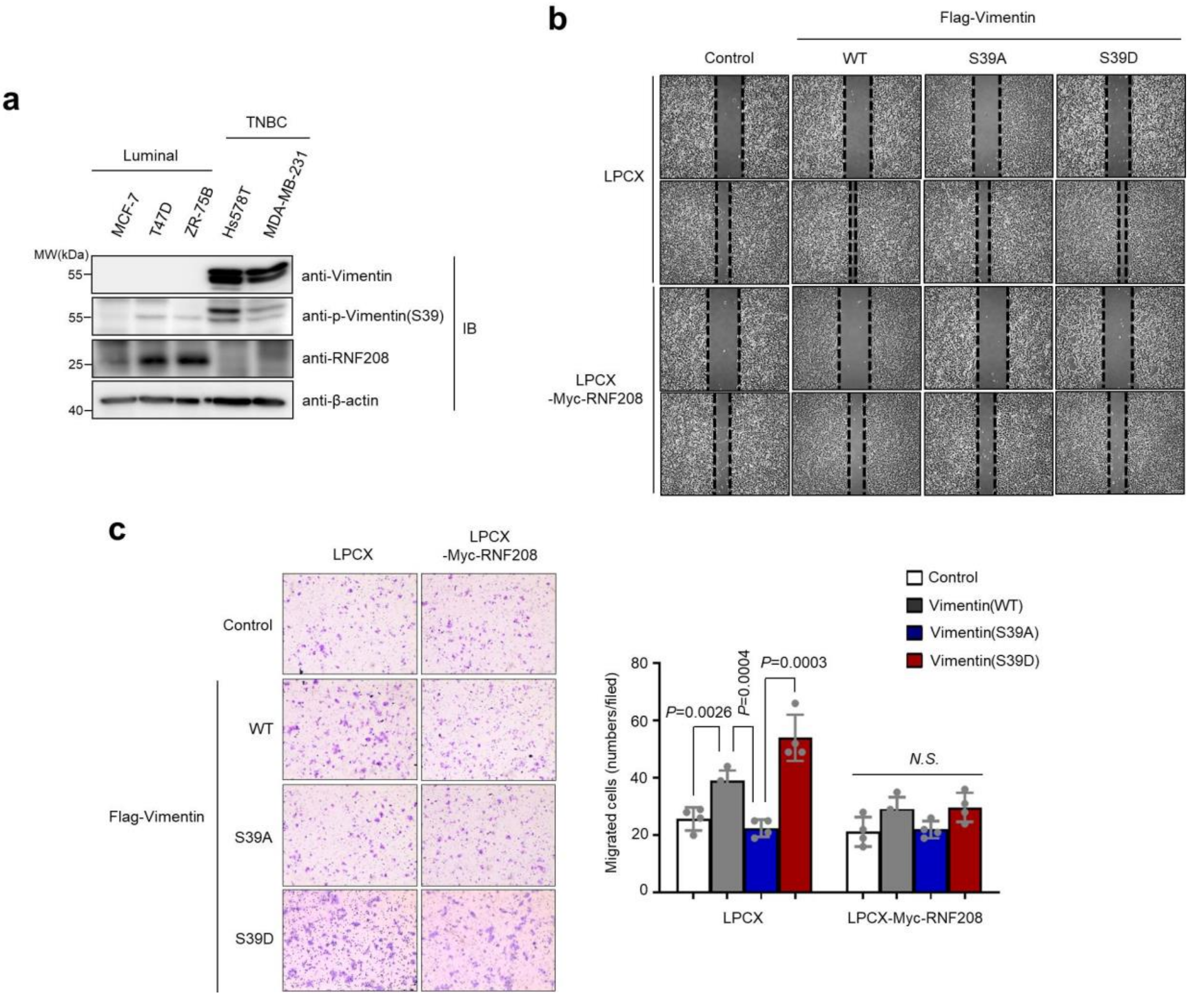

Supplementary Figure 11 RNF208 overexpression decreases the cell migration by targeting the phosphorylation of vimentin at Ser39 residue. (**a**) Immunoblotting showing expression levels of Vimentin, p-Vimentin(S39), and RNF208 in breast cancer cell lines. (**b**) Phase-contrast microscopy images of an *in vitro* wound healing assay at 0 and 24 h after wounding in RNF208-overexpressed MCF-7 cells transfected with Flag-Vimentin WT, S39A, or S39D plasmids, respectively. Original magnification, 100×. (**c**) Transwell migration assays of RNF208-overexpressed MCF-7 cells transfected with Flag-Vimentin WT, S39A, or S39D plasmids, respectively. Migrated cells were counted following staining with crystal violet. *P* value was calculated by unpaired two-tailed Student’s *t*-tests. The data represent means ±S.D. of three independent experiments. Unprocessed original scans of blots in **a** is shown in Supplementary Fig. 13. Source data for **c** is available in Supplementary Table 4.

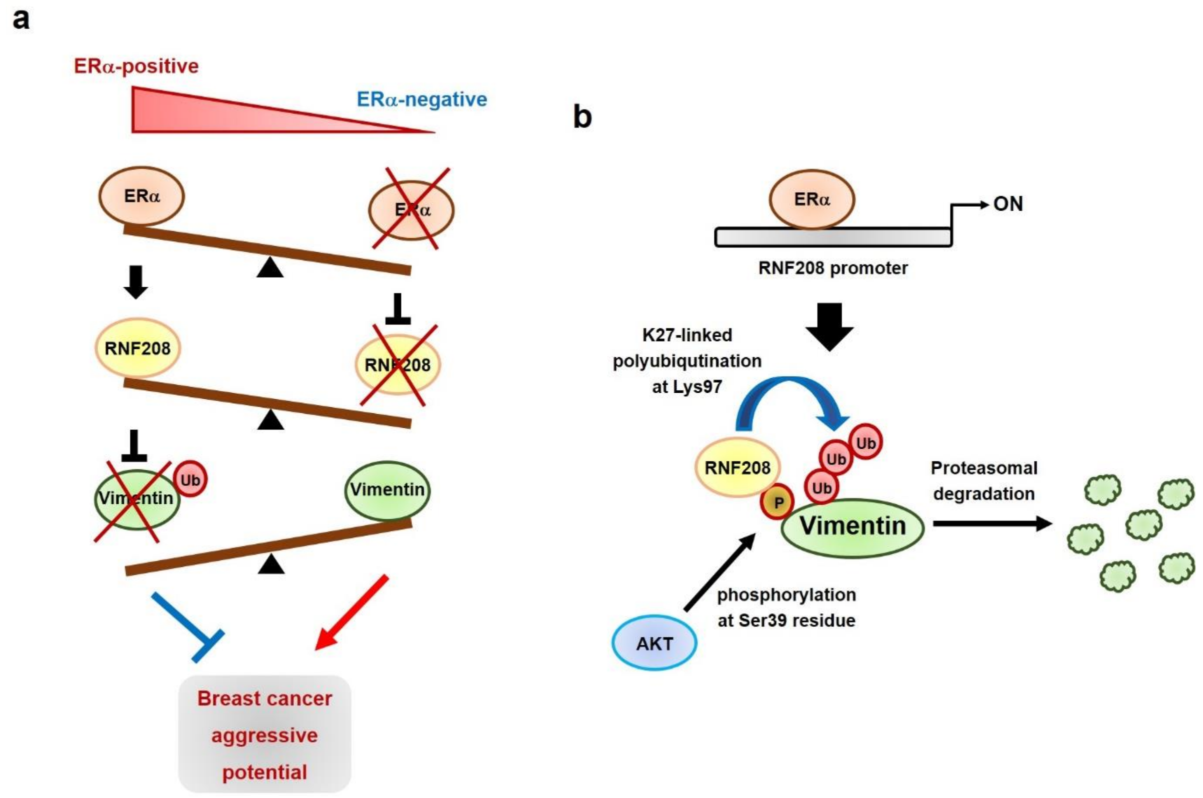

Supplementary Figure 12 Proposed models demonstrating the function of RNF208 as an estrogen-inducible protein in breast cancer progression. (**a**) ERα-dependent expression of RNF208 may block aggressive potential of breast cancer cells by degrading the Vimentin protein. (**b**) Schematic model of the regulation of Vimentin degradation by RNF208. ERα-induced upregulation of RNF208 specifically targets the phosphorylated vimentin at Ser39 as a soluble form for K27-linked polyubiquitination-mediated proteasomal degradation, ultimately suppressing the aggressive breast cancer progression.

Supplementary Figure 13

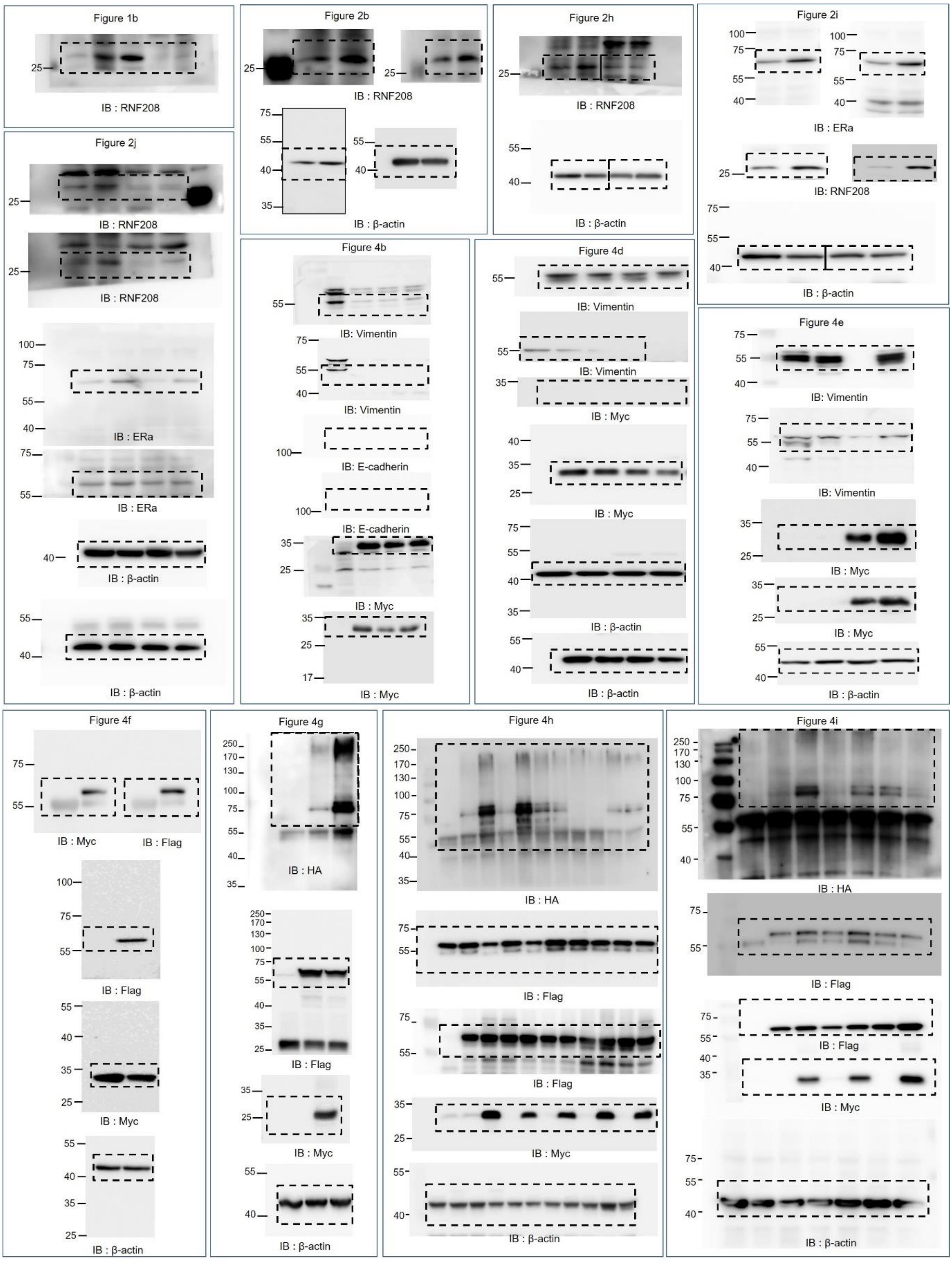

Supplementary Figure 13 Unprocessed original scans of blots.

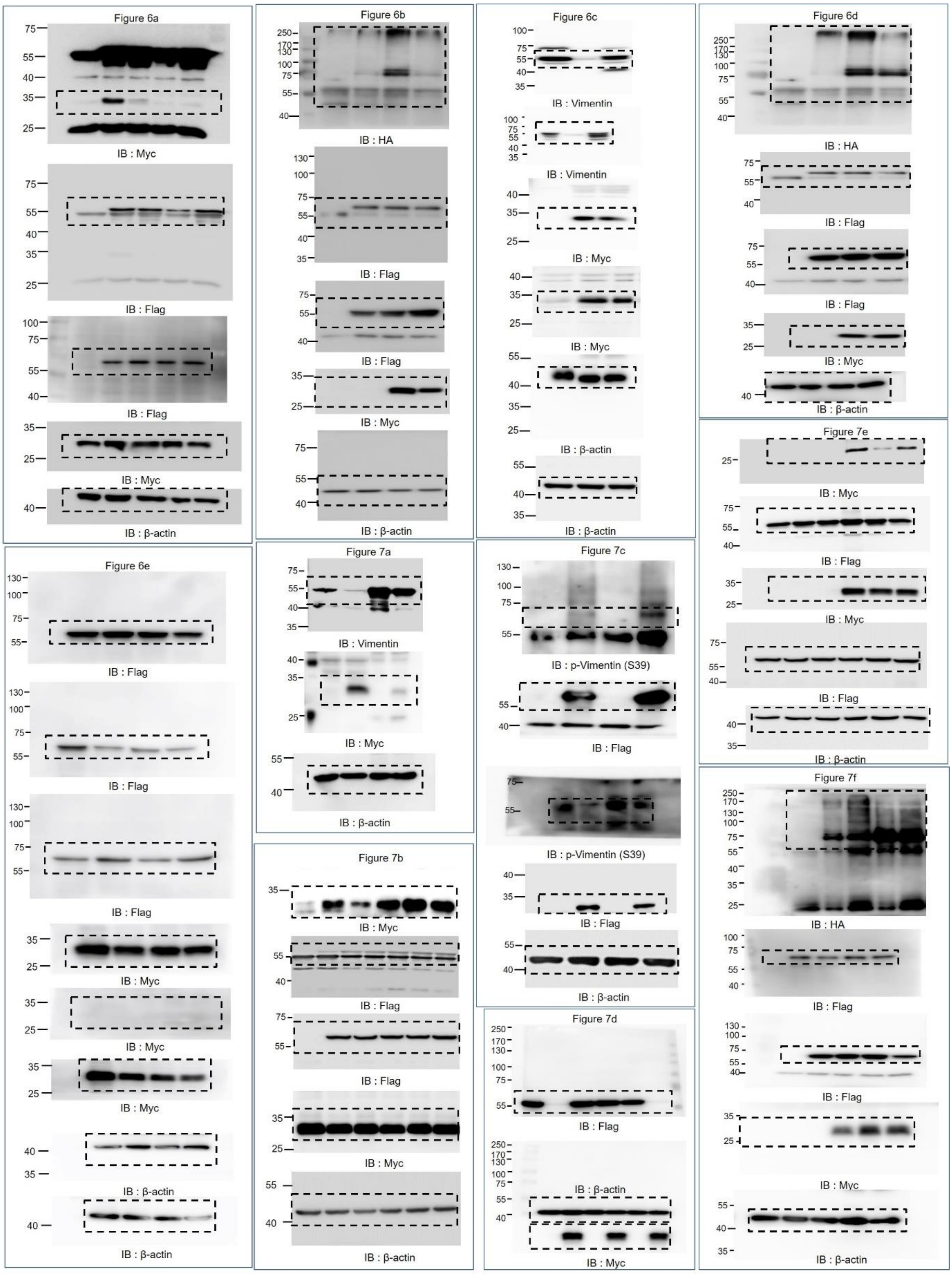

Supplementary Figure 13 Unprocessed original scans of blots.

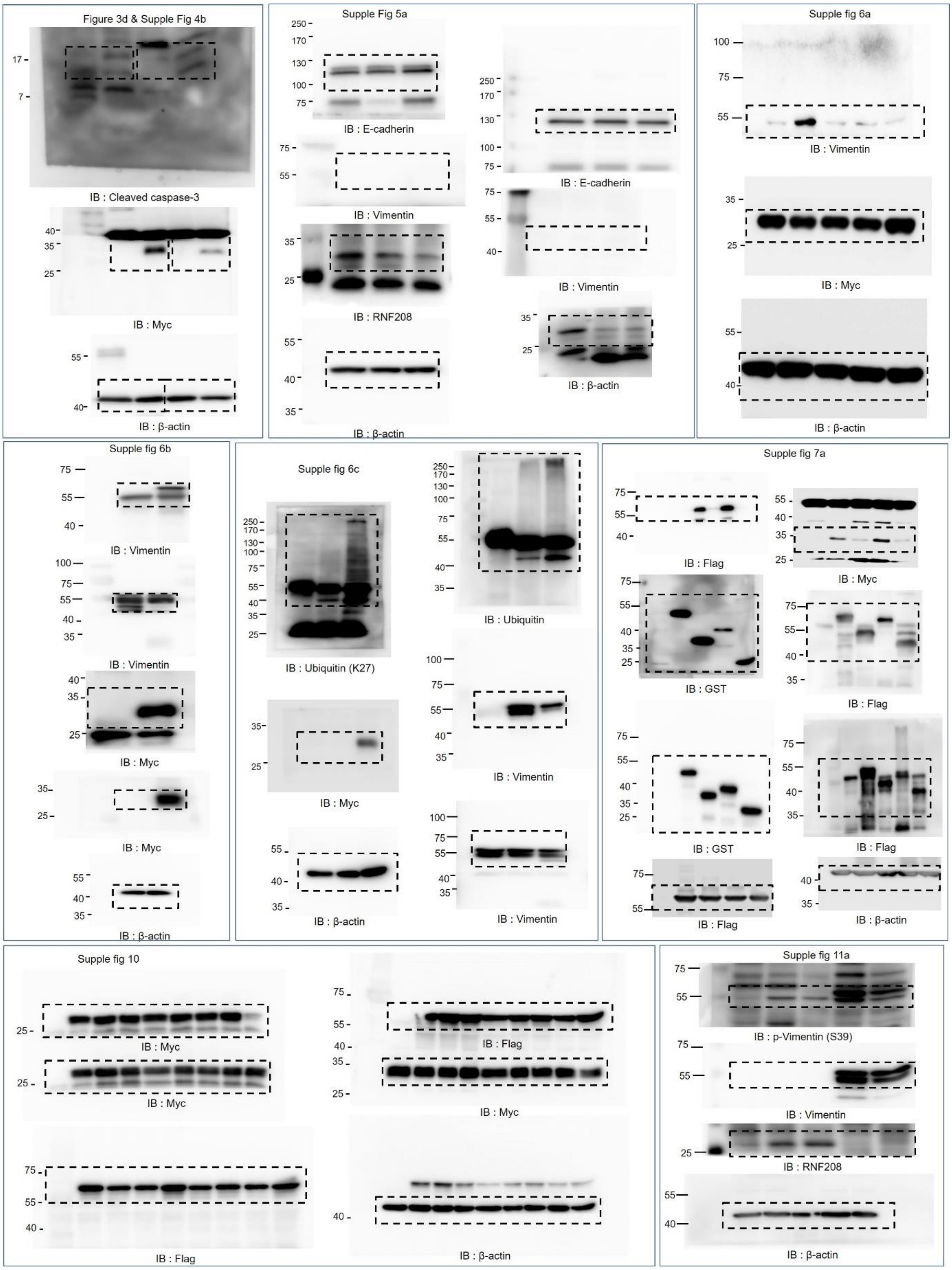

Supplementary Figure 13 Unprocessed original scans of blots.

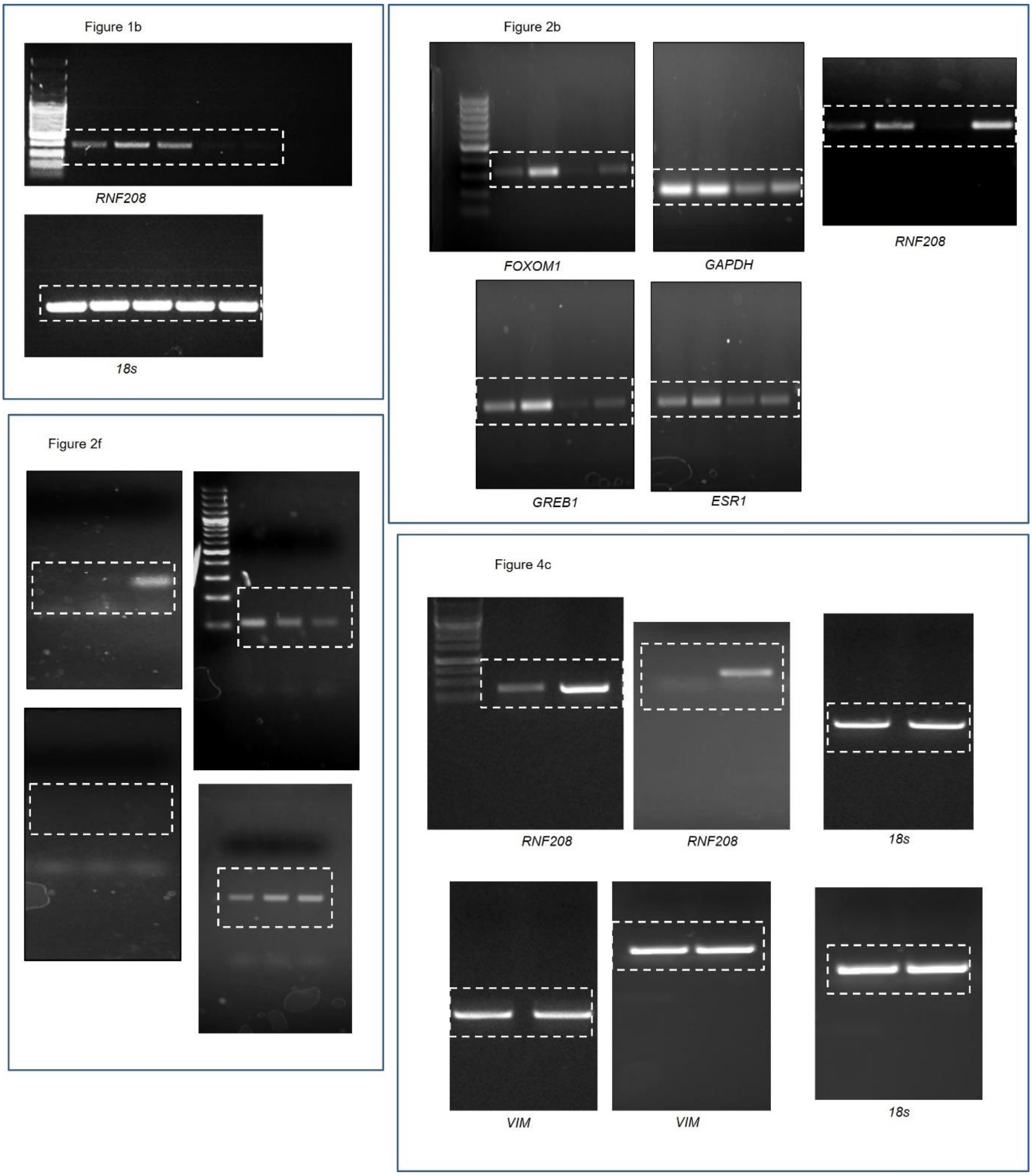

Supplementary Figure 13 Unprocessed original scans of gels.
