# Supplementary material for: RNF208, an estrogen-inducible E3 ligase, targets soluble Vimentin to suppress metastasis in triple-negative breast cancers
Source: Nat Commun. 2019 Dec 20;10:5805. doi: 10.1038/s41467-019-13852-5 (PMC6925134; doi:10.1038/s41467-019-13852-5)
Supplement: Supplementary file 3 — Description of Additional Supplementary Files [file 41467_2019_13852_MOESM3_ESM.pdf]

Description of Additional Supplementary Files

File Name: Supplementary Data 1
Description: List of primer sequences used in this study

File Name: Supplementary Data 2
Description: List of antibodies used in this study

File Name: Supplementary Data 3
Description: List of RNF208 binding partner candidates using mass spectrometry
