# Supplementary material for: RNF208, an estrogen-inducible E3 ligase, targets soluble Vimentin to suppress metastasis in triple-negative breast cancers
Source: Nat Commun. 2019 Dec 20;10:5805. doi: 10.1038/s41467-019-13852-5 (PMC6925134; doi:10.1038/s41467-019-13852-5)
Supplement: Supplementary file 7 — Reporting Summary [file 41467_2019_13852_MOESM7_ESM.pdf]

nature research

Corresponding author(s): Seong-Jin Kim, Kyung-Min Yang

Last updated by author(s): Nov 21, 2019

Reporting Summary

Nature Research wishes to improve the reproducibility of the work that we publish. This form provides structure for consistency and transparency in reporting. For further information on Nature Research policies, see Authors & Referees and the Editorial Policy Checklist.

Statistics

For all statistical analyses, confirm that the following items are present in the figure legend, table legend, main text, or Methods section.

n/a	Confirmed	
☐	☒	The exact sample size (n) for each experimental group/condition, given as a discrete number and unit of measurement
☐	☒	A statement on whether measurements were taken from distinct samples or whether the same sample was measured repeatedly
☐	☒	The statistical test(s) used AND whether they are one- or two-sided<br>*Only common tests should be described solely by name; describe more complex techniques in the Methods section.*
☒	☐	A description of all covariates tested
☐	☒	A description of any assumptions or corrections, such as tests of normality and adjustment for multiple comparisons
☐	☒	A full description of the statistical parameters including central tendency (e.g. means) or other basic estimates (e.g. regression coefficient) AND variation (e.g. standard deviation) or associated estimates of uncertainty (e.g. confidence intervals)
☐	☒	For null hypothesis testing, the test statistic (e.g. F, t, r) with confidence intervals, effect sizes, degrees of freedom and P value noted<br>*Give P values as exact values whenever suitable.*
☒	☐	For Bayesian analysis, information on the choice of priors and Markov chain Monte Carlo settings
☒	☐	For hierarchical and complex designs, identification of the appropriate level for tests and full reporting of outcomes
☒	☐	Estimates of effect sizes (e.g. Cohen's d, Pearson's r), indicating how they were calculated

Our web collection on statistics for biologists contains articles on many of the points above.

Software and code

Policy information about availability of computer code

Data collection	No software was used to collect data for this study.
Data analysis	After the sequencing reaction, low quality reads were filtered according to the following criteria: reads containing more than 10% of skipped bases (marked as 'N's), reads containing more than 40% of bases with quality scores less than 20, and reads having average quality scores where each read is less than 20. The whole filtering process was performed using the in-house scripts. Filtered reads were mapped to the reference genome related to the species using the aligner STAR v.2.4.0b. Gene expression level was measured with Cufflinks v2.1.1 using ensemble 72 database and quantified as the number of mapping reads to a gene divided by the gene length in kilobases and the total number of mapped reads in millions (FPKM).<br>Statistical significance was calculated by using GraphPad Prism 5 and SPSS version 18 software in this study.

For manuscripts utilizing custom algorithms or software that are central to the research but not yet described in published literature, software must be made available to editors/reviewers. We strongly encourage code deposition in a community repository (e.g. GitHub). See the Nature Research guidelines for submitting code & software for further information.

Data

Policy information about availability of data

All manuscripts must include a data availability statement. This statement should provide the following information, where applicable:

- Accession codes, unique identifiers, or web links for publicly available datasets
- A list of figures that have associated raw data
- A description of any restrictions on data availability

RNA-seq data have been deposited in the Gene Expression Omnibus (GEO) database under accession code GSE100878 (Fig. 1a). RNA sequencing datasets for Fig. 1d,e were downloaded from the Genomic Data Common (GDC) data portal (http://portal.gdc.cancer.gov) and deposited public microarray datasets for Fig. 1c,f, Fig. 2a, and Supplementary Fig. 2b are available in the GEO database under accession codes GSE2034, GSE5460, GSE41313, and GSE68379. Relapse-free survival for Fig.

1i and Supplementary Fig. 1 was analyzed by the Kaplan-Meier Plotter analysis tool (http://kmplot.com/analysis). Genomic structure of RNF208 for Supplementary Fig. 2a was obtained from GenBank (https://www.ncbi.nlm.nih.gov/genbank) and TSS database (https://dbtss.hgc.jp).

Field-specific reporting

Please select the one below that is the best fit for your research. If you are not sure, read the appropriate sections before making your selection.

☒ Life sciences ☐ Behavioural & social sciences ☐ Ecological, evolutionary & environmental sciences

For a reference copy of the document with all sections, see nature.com/documents/nr-reporting-summary-flat.pdf

Life sciences study design

All studies must disclose on these points even when the disclosure is negative.

Sample size	No sample-size calculations were performed. Sample size was determined to be adequate based on the magnitude and consistency of measurable differences between groups.
Data exclusions	On principle, data were only excluded for failed experiments, reasons for which included suboptimal activation and microbial contamination.
Replication	All attempts at replication were successful.
Randomization	No randomization of mice. Mice analyzed were litter mates and sex-matched.
Blinding	There were no studies in which investigators were blinded, however results were validated in biological replicates.

Reporting for specific materials, systems and methods

We require information from authors about some types of materials, experimental systems and methods used in many studies. Here, indicate whether each material, system or method listed is relevant to your study. If you are not sure if a list item applies to your research, read the appropriate section before selecting a response.

Materials & experimental systems

n/a	Involved in the study	
☐	☒	Antibodies
☐	☒	Eukaryotic cell lines
☒	☐	Palaeontology
☐	☒	Animals and other organisms
☐	☒	Human research participants
☒	☐	Clinical data

Methods

n/a	Involved in the study	
☒	☐	ChIP-seq
☒	☐	Flow cytometry
☒	☐	MRI-based neuroimaging

Antibodies

Antibodies used	Supplementary Table 2 provided with manuscript contains information on all antibodies used in the study.
Validation	Antibodies used have been used in the literature and also validated by manufacturers.

Eukaryotic cell lines

Policy information about cell lines

Cell line source(s)	The human breast cancer cell lines MCF-7, T47D, Hs578T, and MDA-MB-231 were obtained from the American Type Culture Collection (ATCC); ZR-75B, cloned from the ZR-75-1 cell lines (Dickson et al., 1986), was obtained from the National Cancer Institute (NCI)/NIH.
Authentication	Cell line authentication was confirmed by short tandem repeat (STR)-profiling from ATCC.
Mycoplasma contamination	All cell lines tested negative for mycoplasma contamination.
Commonly misidentified lines (See ICLAC register)	No commonly misidentified lines were used in this study.

Animals and other organisms

Policy information about studies involving animals; ARRIVE guidelines recommended for reporting animal research

Laboratory animals	Mus musculus, NRGA (strain NOD/ShiLtJ-Rag2em1AMC Il2regm1AMC) and NOD/SCID, 6-week-old female
Wild animals	The study did not involve wild animals.
Field-collected samples	The study did not involve samples collected from the field.
Ethics oversight	All procedures were approved the CHA Hospital Animal Care and Use Committee (Seongnam, Korea) and Woojung Bio Animal facility (Suwon, Korea).

Note that full information on the approval of the study protocol must also be provided in the manuscript.

Human research participants

Policy information about studies involving human research participants

Population characteristics	Human breast cancer tissues from surgical section at the Gangnam Severance Hospital, Yonsei University College of Medicine (Seoul, Korea) were collected between January 1996 and December 2004.
Recruitment	The tumor samples were primary tumors collected for diagnosis. These archival tissue blocks were with confirmed diagnosis of breast cancer. The inclusion of tumor tissues was random.
Ethics oversight	Institutional Review Board of Gangnam Severance Hospital, Yonsei University College of Medicine (Seoul, Korea) (IRB approval number 3-2013-0268). All procedures involving human participants were performed in compliance with the relevant ethical standards.

Note that full information on the approval of the study protocol must also be provided in the manuscript.
